# Hsa-miR-874-3p Reduces Endogenous Expression of RGS4-1 Isoform In Vitro

**DOI:** 10.3390/genes15081057

**Published:** 2024-08-11

**Authors:** Feng-Ling Xu, Bao-Jie Wang

**Affiliations:** 1 School of Forensic Medicine, China Medical University, Shenyang 110122, China; xufengling1992@163.com; 2 School of Forensic Medicine, Wannan Medical College, Wuhu 241002, China

**Keywords:** *RGS4* gene, 3′UTR, hsa-miR-874-3p, RGS4-1 isoform, schizophrenia

## Abstract

Background: The level of the regulator of G-protein signaling 4-1 (RGS4-1) isoform, the longest RGS4 isoform, is significantly reduced in the dorsolateral prefrontal cortex (DLPFC) of people with schizophrenia. However, the mechanism behind this has not been clarified. The 3′untranslated regions (3′UTRs) are known to regulate the levels of their mRNA splice variants. Methods: We constructed recombinant pmir-GLO vectors with a truncated 3′ regulatory region of the *RGS4* gene (3R1, 3R2, 3R3, 3R4, 3R5, and 3R6). The dual-luciferase reporter assay was conducted to find functional regions in HEK-293, SK-N-SH, and U87cells and then predicted miRNA binding to these regions. We performed a dual-luciferase reporter assay and a Western blot analysis after transiently transfecting the predicted miRNAs. Results: The dual-luciferase reporter assay found that regions +401–+789, +789–+1152, and +1562–+1990 (with the last base of the termination codon being +1) might be functional regions. Hsa-miR-874-3p, associated with many psychiatric disorders, might target the +789–+1152 region in the 3′UTR of the *RGS4* gene. In the dual-luciferase reporter assay, the hsa-miR-874-3p mimic, co-transfected with 3R1, down-regulated the relative fluorescence intensities. However, this was reversed when the hsa-miR-874-3p mimic was co-transfected with m3R1 (deletion of +853–+859). The hsa-miR-874-3p mimic significantly decreased the endogenous expression of the RGS4-1 isoform in HEK-293 cells. Conclusions: Hsa-miR-874-3p inhibits the expression of the RGS4-1 isoform by targeting +853–+859.

## 1. Introduction

Regulator of G-protein signaling (RGS) proteins regulate G-protein-coupled receptors by activating GTPase, affecting the conduction of signal pathways [1]. To date, 28 RGS proteins have been detected, but among the genes encoding them, only the *RGS4* gene has been widely studied [2]. The *RGS4* gene has been found to be associated with many diseases, namely age-related macular degeneration [3], diet-induced obesity [4], airway hyper-responsiveness [5], and psychiatric illnesses [6]. For example, the expression of this gene, a candidate for involvement in schizophrenia, was found to be significantly changed in the central nervous system postmortem in patients with schizophrenia [7]. In the brain tissues of schizophrenia patients, it is known that the expression of RGS4 is decreased [8,9]. Moreover, Mirnics et al. demonstrated large decreases in RGS4 expression across three cortical areas in subjects with schizophrenia [10]. RGS4 expression deficit in the prefrontal cortex was also found to contribute to behaviors related to schizophrenia in mice [11].

In the brains of humans, five different isoforms, RGS4-1, RGS4-2, RGS4-3, RGS4-4, and RGS4-5, have been identified, with RGS4-1 (the longest splice variant) having the highest expression [12]. It was also reported that the mRNA level of RGS4-1 significantly declined in the dorsolateral prefrontal cortex (DLPFC) of schizophrenia patients compared with that in controls [13]. The levels of the mRNA splice variants are known to be affected by SNPs in the 5′untranslated region (UTR) and 3′UTR [14]. MRNA stability, translational efficiency, and mRNA localization have been reported to be regulated by 3′UTRs [15,16]. The biology of mRNA might also be regulated by cis-elements in the 3′UTRs of genes in cooperation with RNA-binding proteins or microRNA (miRNA) [17].

miRNA, binding with the 3′UTR of the *RGS4* gene, might affect mRNA degradation, splicing, and polyadenylation, causing the 3′UTR of the same gene to produce transcripts of different lengths. It was reported that hsa-miR-124 binds to the 3′UTR of the *RGS4* gene via rs10759C, not rs10759A. rs10759 regulates the expression of the RGS4 protein [18]. However, the regulation of the RGS4-1 isoform has been poorly studied. It was uncovered that miR-107 down-regulates the expression of the *RGS4* gene, influencing liver cell proliferation and invasion, resulting in the development of metabolic disease [19]. It was analyzed that miR-3663 plays an important role in the development of thyroid cancer by adjusting the expression of the *RGS4* gene [20]. A few studies have detected the function of miRNAs in the development of schizophrenia via their binding with the 3′UTR of the *RGS4* gene.

Although the splice variant RGS4-1 exhibits altered expression in schizophrenia, the cis-elements in its 3′UTR remain unclear. We thus conducted this study to detect cis-elements in its 3′UTR.

## 2. Methods

### 2.1. Recombinants Constructed with the 3′ Regulatory Region of the RGS4 Gene

**Figure 1:**
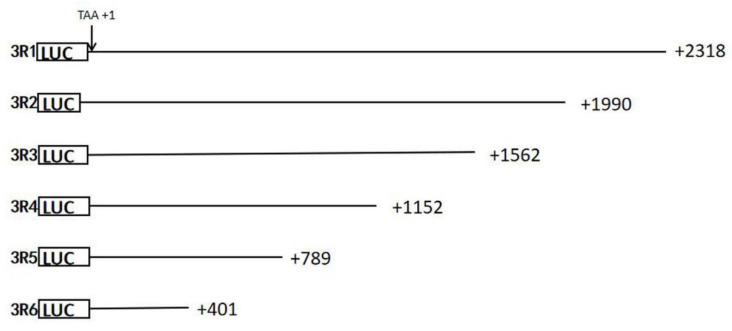
The truncated 3′ regulatory region of the *RGS4* gene recombined into pmir-GLO vectors.

**Figure 2:**
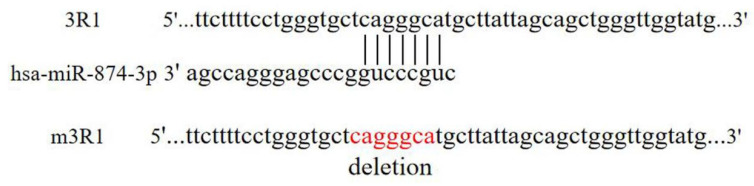
The predicted miR-874-3p binding site in the RGS4 mRNA 3′-UTR and the deletion sequences in m3R1. The red bases are the deleted ones in m3R1.

We selected a DNA sample containing the wild-type 3′UTR of the *RGS4* gene to construct the pmir-GLO (Promega, Madison, WI, USA) recombinant. No more than 8 kinds of vectors were detected in the same 24-well plate. In this study, almost 400bp fragments of the 3′UTR of the *RGS4* gene were deleted one by one, resulting in five kinds of truncated fragments. Fragments of the wild type (3R1) and truncated 3′ regulatory region (3R2, 3R3, 3R4, 3R5, and 3R6) of the *RGS4* gene were amplified using primers with introduced cleavage sites of *NheI* and *XhoI* (Table 1 and Figure 1). Before the target fragments were cloned into pmir-GLO recombinants, these fragments were enriched in the pBM20S vector (Biomed, Beijing, China). Sanger sequencing was performed to verify the cloned fragments. The mutant 3R1 (m3R1) was recombined by General Bio (http://www.generalbio.com/, accessed on 1 July 2024), and the deletion sequence is shown in Figure 2.

### 2.2. Cell Culture and Dual-Luciferase Reporter Assay

There were three kinds of cell lines in this study, namely human embryonic kidney (HEK-293), neuroblastoma (SK-N-SH) and glioblastoma (U87) cells. KeyGEN BioTECH^®^ DMEM (Thermo Fisher Scientific, Waltham, MA, USA) with 10% bovine serum (FBS) was prepared for the HEK-293 and U87 cells, and HyClone^®^ DMEM (Thermo Fisher Scientific, Waltham, MA, USA) with 15% FBS for SK-N-SH. All cells were cultured in an environment with 37 °C and 5% CO_2_.

The cells were cultured to a density of 90% in 24-well plates. The pmir-GLO recombinants (500 ng) were transiently transfected using Lipofectamine^®^2000 reagent (Invitrogen, Carlsbad, CA, USA). A total of 100 ng of recombinant vector 3R1 or m3R1 was co-transfected with 1 μL (20 μM) of the hsa-miR-874-3p mimic or NC with 2 μL of the Lipofectamine^®^2000 reagent (Table 2). Cell lysis for the reporter assay was prepared using the Dual-Luciferase^®^ Reporter Assay System as the protocol, after culturing for 24 h (Promega, Madison, WI, USA). The firefly luciferase activity (LUC) and renilla luciferase activity (TK) were determined in accordance with the manufacturer’s protocol. Each recombinant was tested in triplicate per experiment, with a total of three independent experiments [21].

### 2.3. Bioinformatic Platform Prediction

The miRNAs that bound to the region with significantly decreased expression in the 3′UTR of the *RGS4* gene were predicted with TargetScan Human 7.2 (http://www.targetscan.org/vert_72/, accessed on 1 July 2024). The miRNAs, binding to the functional region, were screened with context++ score percentile ≥95%. The miRNAs which were associated with psychiatric disorders were given priority to be studied.

### 2.4. Western Blotting

Cells placed in six-well plates were transfected with 100 pmol NC, the hsa-miR-874-3p mimic, an NC inhibitor, and an hsa-miR-874-3p inhibitor with 5 μL of Lipofectamine^®^2000 reagent, and the protein was extracted after 72 h (Table 2). The protein levels of the RGS4-1 isoform and β-actin were detected by Western blotting. The total protein was obtained using RIPA with PMSF, and its concentrations was evaluated with a BCA Protein Assay Kit (Beyotime, Shanghai, China). Then, 20 μg of protein in 18 μL total volume was separated on a 12% SDS-PAGE gel and then transferred to a polyvinylidene fluoride membrane for 1 h at 100 V. The membrane was then subjected to immunoblotting with anti-RGS4-1 (H-12) [21] and anti-β-actin antibodies from Santa Cruz Biotechnology on 1:500 dilution (Santa Ana, CA, USA) overnight at 4 °C, after which blocking was performed at room temperature for 2 h in 8% skimmed milk. The protein bands were detected using an enhanced ECL kit in Tanon-5500. The molecular weights of the RGS4-1 and β-actin proteins are 37 and 42 kDa, respectively. The level of protein was presented as a grayscale value using ImageJ software (version 1.8.0) [22].

### 2.5. Statistical Analysis

The relative fluorescence intensity is shown as the value of LUC normalized by TK. Differences in the LUC/TK values between adjacent recombinant vectors were detected by one-way analysis of variance with SPSS 20.0 software (IBM Corp., Armonk, NY, USA) [23]. The differences between the grayscale values were compared using an independent sample t-test with SPSS 20.0 software. A *p*-value less than 0.05 was considered to represent a statistically significant difference.

## 3. Results

### 3.1. Comparison and Analysis of Relative Fluorescence Intensities of Recombined Vectors

**Figure 3:**
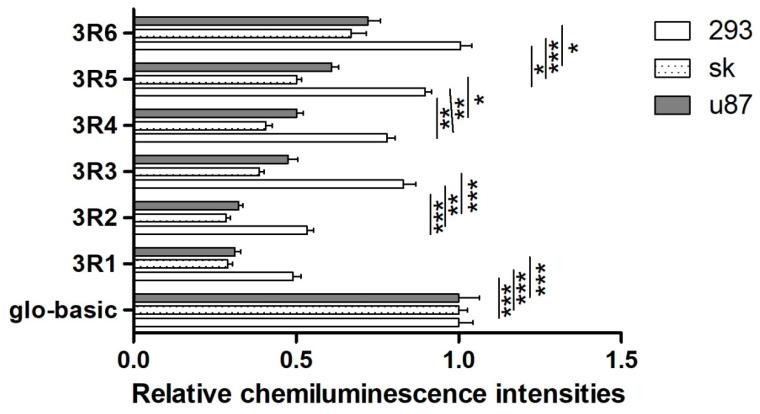
The relative fluorescence intensities of recombined pmir-GLO vectors (3R1–3R6) in HEK-293, U87, and SK-N-SH cells. * *p* ≤ 0.05; ** *p* ≤ 0.01; *** *p* ≤ 0.001.

The relative fluorescence intensities of 3R1 were significantly decreased compared with those of pmirGLO-basic in the three cell lines. To detect the functional region in the 3′ regulatory region of the *RGS4* gene, we determined the relative fluorescence intensities of recombined vectors with truncated fragments (Figure 3). In the HEK-293, SK-N-SH, and U87 cells, significant increases in the relative fluorescence intensities were found in 3R3 compared with those in 3R2 (*p* < 0.001, 95%CI = −0.5823–−0.3170; *p* < 0.01, 95%CI = −0.0253–−0.0049; *p* < 0.01, 95%CI = −0.0725–−0.0167), in 3R5 compared with those in 3R4 (*p* < 0.01, 95%CI = −0.3088–−0.0435; *p* < 0.01, 95%CI = −0.0242–−0.0039; *p* < 0.05, 95%CI = −0.0597–−0.0387), and in 3R6 compared with those in 3R5 (*p* < 0.05, 95%CI = −0.2985–−0.0332; *p* < 0.001, 95%CI = −0.0351–−0.0147; *p* < 0.05, 95%CI = −0.0606–−0.0470). As such, the regions +401–+789, +789–+1152, and +1562–+1990 might contain functional cis-elements.

### 3.2. Results of Bioinformatic Platform Prediction

The miRNAs targeting the sequences of the regions +401–+789, +789–+1152, and +1562–+1990 were predicted in TargetScan Human 7.2. Hsa-miR-874-3p, hsa-let-7f-2-3p and hsa-miR-146b-3p, associated with many psychiatric disorders, might target the functional regions, with a high context++ score percentile. However, hsa-let-7f-2-3p and hsa-miR-146b-3p were poorly expressed in the three cells of this study (Table S1). Hsa-miR-874-3p was selected for further study.

### 3.3. Hsa-miR-874-3p Mimic Inhibited Expression Relative Fluorescence Intensity of 3R1 and Endogenous RGS4-1 Isoform

**Figure 4:**
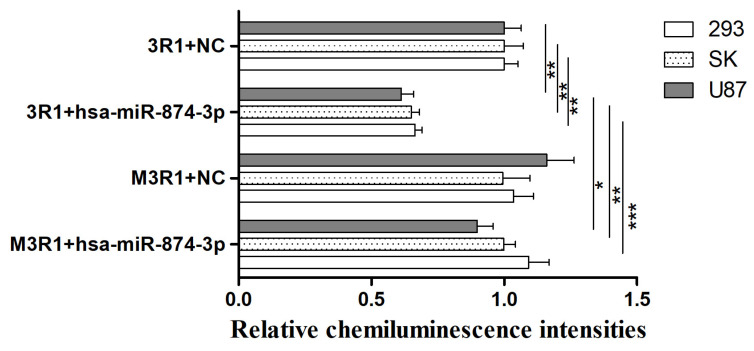
The relative fluorescence intensities of 3R1 and m3R1 co-transfected with hsa-miR-874-3p mimic or NC. * *p* ≤ 0.05; ** *p* ≤ 0.01; *** *p* ≤ 0.001.

Hsa-miR-874-3p might target the sequences of (+853–+859bp), predicted in TargetScan Human 7.2. The mutant 3R1 (m3R1) was recombined by deleting the sequences of (+853–+859bp). Co-transfection of 3R1 or m3R1 with NC or the hsa-miR-874-3p mimic was performed. In the HEK-293, SK-N-SH, and U87 cells, the hsa-miR-874-3p mimic significantly decreased the relative fluorescence intensity of 3R1 compared with NC (*p* < 0.01, 95%CI = 0.0489−0.3016; *p* < 0.01, 95%CI = 0.0050–0.0400; *p* < 0.01, 95%CI = 0.0122–0.0806) (Figure 4). And, the decrease in relative fluorescence intensity for the co-transfection hsa-miR-874-3p mimic with 3R1 was significantly different from the co-transfection hsa-miR-874-3p mimic with m3R1 (*p* < 0.001, 95%CI = −0.3499–0.0972; *p* < 0.01, 95%CI = −0.0400–−0.0049; *p* < 0.05, 95%CI = −0.068–−0.0001).

**Figure 5:**
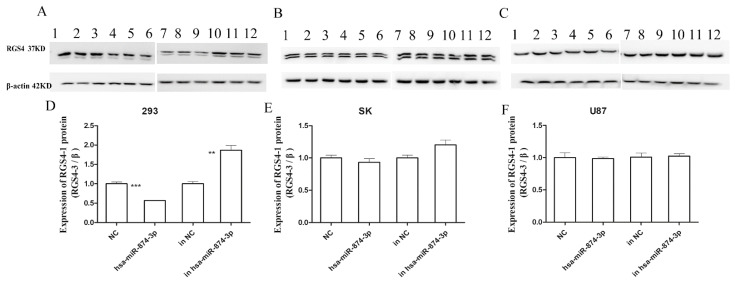
Western blot assay measuring the endogenous protein levels of the RGS4-1 inform after transfection of NC (1, 2, 3), the hsa-miR-874-3p mimic (4, 5, 6), an NC inhibitor (7, 8, 9), and an hsa-miR-874-3p inhibitor (10, 11, 12), in HEK-293 (**A**,**D**), SK (**B**,**E**), and U87 (**C**,**F**). ** *p* ≤ 0.01; *** *p* ≤ 0.001, (**A**–**C**) each panel summarizes data from two separate western blots.

In the HEK-293 cell line, Western blotting showed that the hsa-miR-874-3p mimic significantly negatively regulated the endogenous expression of the RGS4-1 isoform compared with the case with NC (*p* < 0.001, 95%CI = 0.3093–0.5601) (Figure 5). And, the hsa-miR-874-3p inhibitor significantly increased the expression of the RGS4-1 isoform than inhibitor NC (*p* < 0.01, 95%CI = −1.236–−0.4934) in Figure 5.

## 4. Discussion

The 3′UTR of the *RGS4* gene negatively regulates its expression. The regions +401–+789, +789–+1152, and +1562–+1990 might serve a function in this, as revealed by the dual-luciferase reporter assay in our study. The binding sites for hsa-miR-874-3p in the +789–+1152 regions were identified. The reduced expression of 3R1 disappeared in m3R1 upon deletion of the binding site at the +853–+859 region. The level of the RGS4-1 protein was significantly reduced in the HEK-293 cell line treated with hsa-miR-874-3p compared with that treated with NC. Hsa-miR-874-3p negatively regulated the endogenous expression of the RGS4-1 isoform.

It was revealed that alternative mRNA isoforms are regulated by differences in the 3′UTR, and the neural mRNAs appear to express significantly longer 3′UTRs [24,25]. MiRNA, binding to 3′UTR cis-elements, was also reported to regulate the expression of genes and diverse fates of mRNAs [26]. MiRNA was also found to participate in the miRNA-directed translational repression and inhibition of translational initiation and poly(A) shortening, resulting in mRNA destabilization [27]. It was reported that hsa-miR-874-3p is a potential therapeutic target for many diseases, such as hepatic cellular carcinoma [28], pancreatic cancer, lung cancer [29], osteosarcoma [30], and major depressive disorder [31]. MiR-874-3p was found to be increased in both neurons and microglia, taking part in inhibiting depression-like behavior. Hsa-miR-874-3p, targeting the *RGS4* gene (+603–+631), was found to inhibit cell migration in osteosarcoma [30].

Another hsa-miR-874-3p target site (+853–+859) in the *RGS4* gene was predicted and confirmed in our study. RGS4-1, the longest RGS4 isoform, was reported to be specifically decreased in the DLPFC of schizophrenia patients [14]. Anti-RGS4-1 (H-12) was shown to specifically identify the RGS4-1 isoform. In the present study, the hsa-miR-874-3p mimic down-regulated the expression of the RGS4-1 isoform by targeting +853–+859 in the 3′UTR in the HEK-293 cells. Moreover, the hsa-miR-874-3p inhibitor increased the expression of the RGS4-1 isoform in the HEK-293 cells. Hsa-miR-874-3p down-regulated the expression of the RGS4-1 isoform protein in the HEK-293 cells, but not in the SK-N-SH and U87 cells. These inconsistent results might be due to the different microenvironments of the different cell types, which in turn affected the interaction between the miRNA and target genes [32].

There were some limitations in this study. First, the miRNAs, binding with other functional regions of +789–+1152 and +1562–+1990 in the 3′UTR of the *RGS4* gene, were not analyzed. Second, an animal study was not performed to detect the function of hsa-miR-874-3p in the expression of the RGS4-1 isoform. Third, the cooperation of circRNA/miRNA was not detected in this study. Therefore, in a further study, an animal study should be performed and the cooperation of circRNA/hsa-miR-874-3p should be studied to detect the function of hsa-miR-874-3p on the expression of the RGS4-1 isoform.

In conclusion, the regions +401–+789, +789–+1152, and +1562–+1990 in the 3′UTR of the *RGS4* gene might be functional regions. The hsa-miR-874-3p target site (+853–+859) in the 3′UTR of the *RGS4* gene was predicted and confirmed in this study. Hsa-miR-874-3p was shown to inhibit the endogenous expression of the RGS4-1 isoform. It should be noted that the function of Hsa-miR-874-3p in the expression of the RGS4-1 isoform needs further animal and circRNA studies. The other functional fragments (+401–+789 and +1562–+1990) might bind other trans-acting elements, and that also needs further study.

## Figures and Tables

**Table 1:** Primers for construction of recombinant pmir-GLO vectors.

Primer	Sequences
3RF	5′ CTAGCTAGCTGCTTCCCTGGTCC 3′
3RR1	5′ CCCTCGAGATTTTTCTAAAGTCACC 3′
3RR2	5′ CCCTCGAGGACCTATTAGTAAGACAGC 3′
3RR3	5′ CCCTCGAGAGGTGATATCCTGTACC 3′
3RR4	5′ CCCTCGAGAGCCTCATCTGATGG 3′
3RR5	5′ CCCTCGAGAGCATGAAGTTCCTTG 3′
3RR6	5′ CCCTCGAGTCTGGAGAACGTGC 3′

The underlined bases were the introduced cleavage sites.

**Table 2:** Sequences of miRNA.

Primer		Sequences
hsa-miR-874-3p mimics	5′-3′	CUGCCCUGGCCCGAGGGACCGA
		GGUCCCUCGGGCCAGGGCAGUU
hsa-miR-874-3p inhibitor	5′-3′	UCGGUCCCUCGGGCCAGGGCAG
NC	5′-3′	UUCUCCGAACGUGUCACGUTT
		TTAAGAGGCUUGCACAGUGCA
NC inhibitor	5′-3′	CAGUACUUUUGUGUAGUACAA

## Data Availability

This was an evidence synthesis study; all data are presented in the Supplementary Materials.

## Supplementary Materials

The following supporting information can be downloaded at: https://www.mdpi.com/article/10.3390/genes15081057/s1, Table S1. The expression of miRNA in the three cell lines.

## Abbreviations

RGS: regulator of G-protein signaling; DLPFC: dorsolateral prefrontal cortex; UTR: untranslated region; miRNA: microRNA; LUC: firefly luciferase activity; TK: renilla luciferase activity.
