# Supplementary material for: Hsa-miR-874-3p Reduces Endogenous Expression of RGS4-1 Isoform In Vitro
Source: Genes (Basel). 2024 Aug 11;15(8):1057. doi: 10.3390/genes15081057 (PMC11353307; doi:10.3390/genes15081057)
Supplement: Supplementary file 1 [file genes-15-01057-s001.zip › genes-3079112-supplementary.pdf]

Table S1. The expression of miRNA in the three cell lines.

MiRNA	Indicators and internal parameters	293 (CT)	U87 (CT)	SK (CT)
hsa-miR-874-3p	miRNA	24.259	25.395	27.468
	miRNA	24.126	25.459	27.816
	U6	11.492	12.728	13.873
	U6	11.438	12.512	13.760
hsa-let-7f-2-3p	miRNA	>40.000	>40.000	35.834
	miRNA	>40.000	>40.000	35.358
	U6	10.763	11.463	11.746
	U6	10.757	11.330	11.385
hsa-miR-146b-3p	miRNA	31.382	33.504	34.773
	miRNA	32.487	33.942	35.056
	U6	10.173	11.039	11.051
	U6	9.933	10.883	11.036
